# Development of SNP, KASP, and SSR Markers by BSR-Seq Technology for Saturation of Genetic Linkage Map and Efficient Detection of Wheat Powdery Mildew Resistance Gene *Pm61*

**DOI:** 10.3390/ijms20030750

**Published:** 2019-02-11

**Authors:** Jinghuang Hu, Jingting Li, Peipei Wu, Yahui Li, Dan Qiu, Yunfeng Qu, Jingzhong Xie, Hongjun Zhang, Li Yang, Tiantian Fu, Yawei Yu, Mengjuan Li, Hongwei Liu, Tongquan Zhu, Yang Zhou, Zhiyong Liu, Hongjie Li

**Affiliations:** 1 The National Engineering Laboratory of Crop Molecular Breeding, Institute of Crop Sciences, Chinese Academy of Agricultural Sciences, Beijing 100081, China; hujh2016@163.com (J.H.); ml3051378331_2@163.com (P.W.); 13522357314@163.com (Y.L.); smart_qd@163.com (D.Q.); zhanghongjun01@caas.cn (H.Z.); yangli02@caas.cn (L.Y.); liuhongwei@caas.cn (H.L.); zhouyang@caas.cn (Y.Z.); 2 College of Chemistry and Environment Engineering, Pingdingshan University, Pingdingshan 467000, China; 15936057197@163.com (T.F.); 776117360@139.com (Y.Y.); mengjuan9768@163.com (M.L.); 3 College of Life Science and Technology, Harbin Normal University, Harbin 150080, China; quyunfeng1993@163.com; 4 Institute of Genetics and Developmental Biology, Chinese Academy of Sciences, Beijing 100101, China; jzxie@genetics.ac.cn; 5 Zhumadian Academy of Agricultural Sciences, Zhumadian 463000, China; zmdwheat@163.com

**Keywords:** *Triticum aestivum*, Landrace, Powdery mildew, Bulked segregant analysis-RNA-Seq (BSR-Seq), Single nucleotide polymorphism (SNP), Kompetitive Allele Specific PCR (KASP)

## Abstract

The gene *Pm61* that confers powdery mildew resistance has been previously identified on chromosome arm 4AL in Chinese wheat landrace Xuxusanyuehuang (XXSYH). To facilitate the use of *Pm61* in breeding practices, the bulked segregant analysis-RNA-Seq (BSR-Seq) analysis, in combination with the information on the Chinese Spring reference genome sequence, was performed in the F_2:3_ mapping population of XXSYH × Zhongzuo 9504. Two single nucleotide polymorphism (SNP), two Kompetitive Allele Specific PCR (KASP), and six simple sequence repeat (SSR) markers, together with previously identified polymorphic markers, saturated the genetic linkage map for *Pm61*, especially in the proximal side of the target gene that was short of gene-linked markers. In the newly established genetic linkage map, *Pm61* was located in a 0.71 cM genetic interval and can be detected in a high throughput scale by the KASP markers *Xicsk8* and *Xicsk13* or by the standard PCR-based markers *Xicscx497* and *Xicsx538*. The newly saturated genetic linkage map will be useful in molecular marker assisted-selection of *Pm61* in breeding for disease resistant cultivar and in its map-based cloning.

## 1. Introduction

Powdery mildew is one of the most widely epidemic diseases in wheat (*Triticum aestivum* L.) grown in the temperate and humid regions of the world. The causal agent of powdery mildew, *Blumeria graminis* f. sp. *tritici* (*Bgt*), is an obligate biotrophic fungus, which usually colonizes wheat leaves and develops white pustule symptoms on leaf blades. The penalty in wheat yield caused by infection of powdery mildew has been reported to be from 5% to 40% in various countries, depending on the disease severity [1]. The impact of powdery mildew on grain quality, such as test weight and protein content, has been reported [2]. Changes in grain proteome and composition and grain starch and protein contents caused by the disease were observed using the proteomics analysis [3].

In China, powdery mildew has become an economically important disease since the 1970s. In recent years, the annual areas of powdery mildew infected wheat fields ranged from 6 to 8 million hectares in most winter wheat and parts of spring wheat fields throughout the country (available online: https://www.natesc.org.cn/sites/cb/). Management of wheat powdery mildew relies on growing disease resistant cultivars in accompany with application of fungicides such as triadimefon when necessary.

Breeding for powdery mildew resistant cultivars necessitates the availability of powdery mildew resistance genes (*Pm* genes). Sources of *Pm* genes include common wheat and its close or distant relative species. Wheat landraces from China are a class of historically grown and maintained common wheat cultivars, which have provided quite a number of *Pm* genes. A group of landraces carry allelic genes in the *Pm5* locus on chromosome 7BL, for example, *Pm5d* (IGV1-455) [4], *Pm5e* (Fuzhuang 30) [5], *PmH* (Hongquanmang) [6], *PmTm4* (Tangmai 4) [7], *Mlmz* (Mazhamai) [8], *Mlxbd* (Xiaobaidong) [9], *pmHYM* (Hongyoumai) [10], *PmBYYT* (Baiyouyantiao) [11], and *PmSGD* (Shangeda) [12]. In the *Pm24* locus, there are two alleles, *Pm24a* in Chiyacao [13] and *Pm24b* in Baihulu [14]. The designated genes *Pm2c* on 5DS [15], *Pm45* on 6DS [16], and *Pm47* on 7BS [17] were identified in Niaomai, Wuzhaomai, and Hongyanglazi, respectively. *PmX* in Xiaohongpi [18] and *MlHLT* in Hulutou [19] were detected on chromosomes 2AL and 1DS, respectively. Recently, a group of scientists in the USA characterized three *Pm* genes in wheat landraces: two from Afghanistan, *Pm223899* on 1AS in PI 223,899 [20] and *Pm59* on 7AL in PI 181,356 [21], and the other one from Iran, *Pm63* on 2BL in PI 628,024 [22].

Landraces of wheat are traditionally grown in agriculture until they have been replaced by more productive cultivars since the initiation of modern crop breeding in the mid-20th century. At present, landraces can only be grown in certain marginal lands with less productivity [23]. They are no longer adapting to most of the improved agricultural environments in spite of possessing desirable genes. There is a need to introgress the useful genes, for example, *Pm* genes, into modern improved genetic backgrounds in order to be used efficiently in modern breeding practices. Molecular approaches facilitate identification and transfer of wheat genes for disease resistance [24]. In fact, many recently characterized *Pm* genes are identified with the aid of various classes of molecular markers, such as SSR (microsatellite) markers and STS (sequence-tagged site) markers, which are useful in marker-assisted selection (MAS) of target genes.

The breeder-friendly molecular markers associated with resistance genes are useful in the breeding programs during development of disease resistant cultivars. The PCR-based markers are affordable in most wheat breeding programs, so this type of molecular markers can be routinely used in breeding practice. Moreover, high-throughput genotyping is needed in a large scale of population study. The newly improved next-generation sequencing techniques allow the discovery of numerous single nucleotide polymorphism (SNP) markers. The Chinese Spring wheat reference genome sequence has been updated [25], which facilitates the identification of gene-linked molecular markers and map-based cloning of disease resistance genes. The abundance of SNP markers is far greater than the traditional PCR-based markers. The SNP markers can be visualized by converting them into Kompetitive Allele Specific PCR (KASP) markers for establishing high-throughput genotyping platform for MAS of the target genes [26]. They have also been used to detect disease resistance genes, such as *Sr26* for resistance to stem rust (caused by *Puccinia graminis* f. sp. *tritici*) [27], *Yr34* and *Yr48* for resistance to stripe rust (caused by *Puccinia striiformis* f. sp. *tritici*) [28].

BSR-seq technique, which integrates bulked segregant analysis and RNA-seq [29], has proven to be a rapid and efficient strategy to identify gene-linked molecular markers. It provides a fast and high-throughput method to localize resistance genes in crops with large genome, e.g., wheat. This technique has been used in the molecular characterization of wheat disease resistance genes, such as *Yr15* [30], *YrZH22* [31], *YrMM58* and *YrHY1* [32], *Yr26* [33], *Pm4b* [34], and *PmSGD* [12].

Wheat landrace Xuxusanyuehuang (XXSYH) was resistant to several *Bgt* isolates from China, and a recessive gene, *Pm61*, was located on chromosome 4AL [35]. In the genetic linkage map that was developed based on the mapping population of XXSYH × Mingxian 169, *Pm61* was mapped in a 0.46 cM genetic interval on 4AL. However, only two linked markers were identified in the proximal side of *Pm61*. Taking the advantage of BSR-seq and the Chinese Spring reference genome sequence, this study was conducted to (1) saturate genetic linkage map for *Pm61*, and (2) develop PCR-based markers for breeder-friendly use and KASP markers for large scale and high-throughput detection of *Pm61* during its MAS.

## 2. Results

### 2.1. Evaluation of Resistance to Bgt Isolates in XXSYH

Thirty *Bgt* isolates collected from wheat fields in Shandong, Shanxi, Beijing, Hebei, and Sichuan provinces were used to test response of XXSYH to powdery mildew. Twenty isolates were avirulent on XXSYH with ITs 0, 0; 1 or 2. Ten isolates were virulent on XXSYH with ITs 3 or 4 (Table 1). All the *Bgt* isolates tested were virulent on Zhongzuo 9504, the susceptible control.

### 2.2. Genetic Analysis of Powdery Mildew Resistance in XXSYH

**Figure 1:**
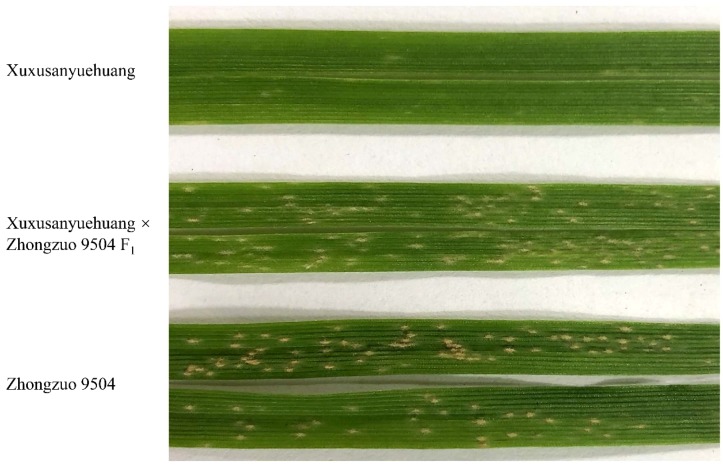
The phenotypic reactions of resistant parent Xuxusanyuehuang, susceptible parent Zhongzuo 9504, and their F_1_ plants to isolate *Bgt1*.

When inoculated with isolate *Bgt1* from Shandong, province, XXSYH was resistant with an IT 1, while Zhongzuo 9504 was susceptible with an IT 3. Therefore, this *Bgt* isolate was able to differentiate the phenotypes of the two parents that were crossed to develop the populations for genetic analysis. The IT of the 15 F_1_ plants from the XXSYH × Zhongzuo 9504 resembled the susceptible parent Zhongzuo 9504 (Figure 1). The 211 F_2:3_ lines produced 51 homozygous resistant, 115 heterozygous, and 45 homozygous susceptible lines, which agrees to the 1:2:1 segregating ratio (χ^2^ = 2.05, *P* = 0.3584). This indicates that the resistance of XXSYH to isolate *Bgt1* was in accordance with the single recessive mode of inheritance.

### 2.3. BSR-Seq Analysis of the Bulked RNA Pools with Distinct Phenotypes to Isolate Bgt1

**Figure 2:**
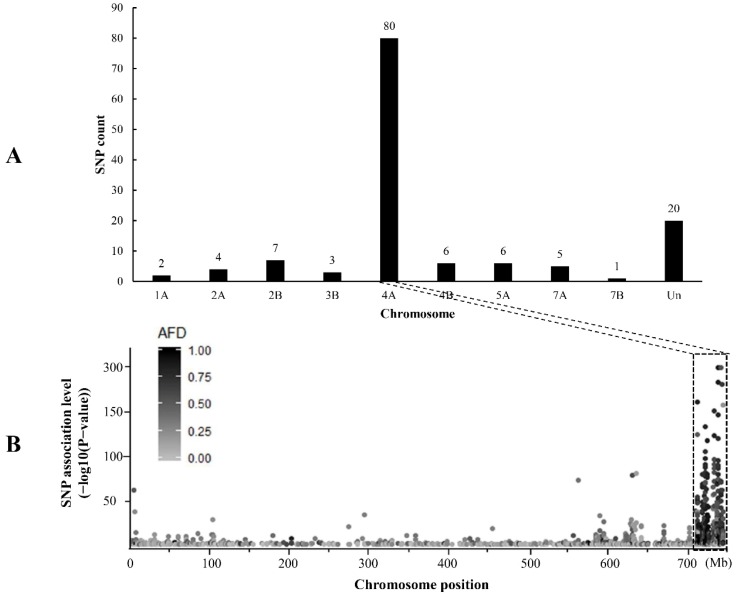
Number of polymorphic single nucleotide polymorphism (SNP) distributed on different wheat chromosomes (**A**) and distribution of SNP variants on chromosome 4A (**B**).

RNA-seq analysis generated a total of 40.0 Gb of raw data from the resistant and susceptible RNA samples and 38.3 Gb (95.77%) of clean data were obtained after quality control. Among the 62,760,214 and 67,764,274 high-quality reads for the two bulks, 58,213,973 (92.76%) and 62,295,839 (91.93%) were uniquely mapped to the Chinese Spring reference genome sequence, respectively (Supplementary Table S1). With the criteria of *P* < 1 × 10^−10^ and AFD > 0.6, 134 SNP variants, potentially associated with the target powdery mildew resistance gene, were identified. Further analysis indicated that 80 (59.7%) candidate SNP were distributed on chromosome 4AL (Figure 2A) and corresponded to a 31 Mb interval in the terminal region of 4AL in the reference genome (Figure 2B). This is consistent with previous study in which *Pm61* was mapped in a 1.3 Mb genomic region (717,963,176–719,260,469) on chromosome 4AL [35].

### 2.4. Validation of the Candidate SNP and Development of SNP Markers

**Figure 3:**
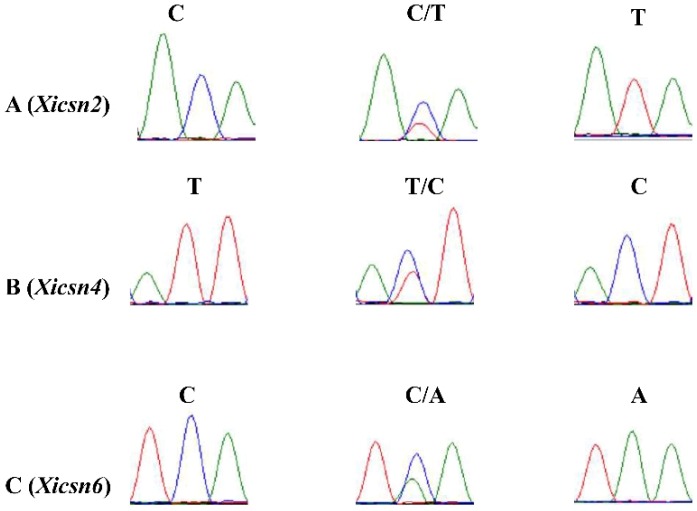
Sanger sequencing profiles of SNP markers *Xicsn2* (**A**), *Xicsn4* (**B**) and *Xicsn6* (**C**) in the homozygously resistant (R), homozygously susceptible (S), and heterozygous F_2:3_ lines (H) from the mapping population of the Xuxusanyuehuang × Zhongzuo 9504 cross. Blue, green, and red lines represent bases of cytosine (C), adenine (A), and thymine (T), respectively.

Using the sequences flanking the 80 SNP that were anchored on chromosome 4AL as the queries, Blast analysis against the Chinese spring whole genome assembly (available online: https://urgi.versailles.inra.fr/) produced 24 homologous scaffolds. The 3 kb sequences containing the candidate SNP and corresponding to the above homologous scaffold were used as templates to design 28 pairs of SNP primers on the GSP website (Supplementary Table S2). The amplified products from XXSYH, Zhongzuo 9504, and the resistant and susceptible DNA bulks were sequenced for polymorphism analysis, and seven SNP markers were polymorphic. Based on the linkage analysis with 16 randomly selected F_2:3_ lines, *Xicsn1*, *Xicsn2*, and *Xicsn3* were potentially mapped on one side of *Pm61*, and *Xicsn4*, *Xicsn5*, *Xicsn6*, and *Xicsn7*, on the other side of target gene. Sequencing analysis of *Xicsn2* and *Xicsn4* was carried out in 211 F_2:3_ lines (Figure 3). *Pm61* was localized in a 4.5 cM genetic interval between the SNP markers *Xicsn2* and *Xicsn4* corresponding to a 5.3 Mb physical region (713,523,186–718,866,838) on the distal end of chromosome 4AL. The other five SNP markers were not used in genotyping the mapping population because of poor clustering of the fluorescence signals.

### 2.5. Development of KASP Markers 

**Figure 4:**
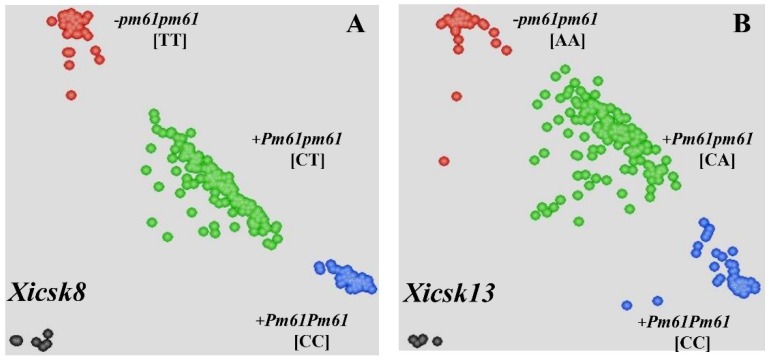
Genotyping results of *Xicsk8* (**A**) and *Xicsk13* (**B**) by Kompetitive Allele Specific PCR (KASP) assay. The scatter plot with the axes *x* and *y* represents the allelic discrimination of *Xicsk8* or *Xicsk13* genotypes. The red, green and blue dots represent the homozygously susceptible, heterozygous, and homozygously resistant F_2:3_ lines from the mapping population of the Xuxusanyuehuang × Zhongzuo 9504 cross, respectively.

To cost-effectively detect *Pm61* in MAS, 15 SNP generated from BSR-Seq analysis between the SNP markers *Xicsn1* and *Xicsn7* were converted into 13 KASP primer pairs (Supplementary Table S3). They were subjected to polymorphism analysis on the parental cultivars and 15 F_2:3_ lines (including five homozygous resistant, heterozygous, and homozygous susceptible lines each). Six polymorphic KASP markers, i.e., *Xicsk4*, *Xicsk5*, *Xicsk7*, *Xicsk8*, *Xicsk9*, and *Xicsk13*, were identified. Moreover, the amplicons of 31 SSR primer pairs, which were previously designed based on the genomic sequence corresponding to the genetic interval between markers *Xicsn2* and *Xicsn4*, were sequenced to detect the SNP variants that differed between the two parents. Seven SNP variants were identified in the amplicons of 7 SSR primer pairs and were converted into KASP primer pairs (Supplementary Table S3). The KASP markers *Xicsk14* and *Xicsk15* showed clear polymorphism between the two parents and 15 F_2:3_ lines by sequencing analysis. By genotyping the F_2:3_ mapping population, KASP markers *Xicsk8* and *Xicsk13* were linked to *Pm61* (Figure 4A,B).

### 2.6. Development of SSR Markers

**Figure 5:**
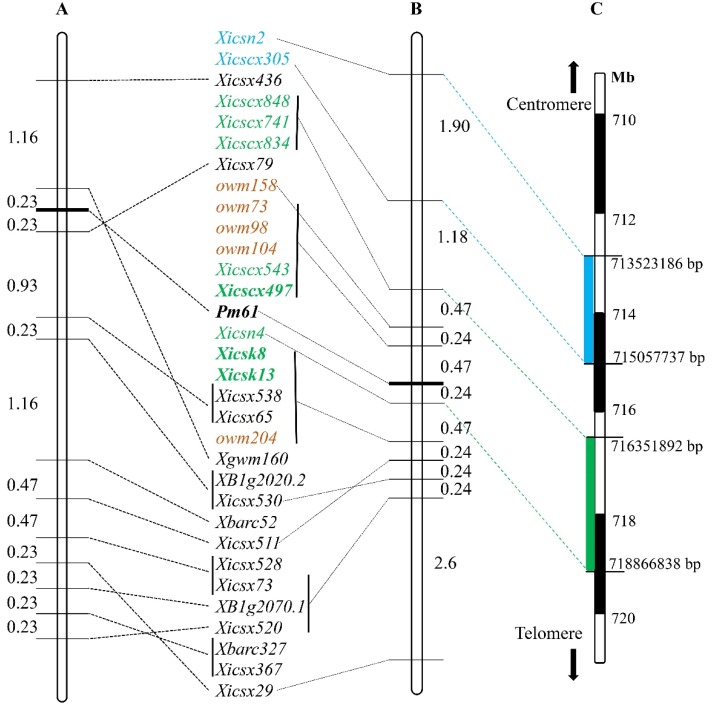
Genetic linkage maps of *Pm61* in previous study [35] (**A**) and newly developed in the present study (**B**). The positions of the *Pm61*-linked molecular markers are indicated on a scale bar based on the Chinese Spring genome sequence (**C**).

The polymorphism of the eleven SSR markers, previously linked to *Pm61* using the mapping population of XXSYH × Mingxian 169 (Figure 5A), was analyzed against the mapping population of XXSYH × Zhongzuo 9504. Seven SSR markers, *Xicsx29*, *Xicsx65*, *Xicsx73*, *Xicsx511*, *Xicsx520*, *Xicsx530*, and *Xicsx538*, were polymorphic. Linkage analysis indicated that all of these polymorphic markers were located on the distal side of *Pm61* (Figure 5B). Markers *Xicsx79* and *Xicsx436* previously located in the proximal side of *Pm61* were not polymorphic in the XXSYH × Zhongzuo 9504 mapping population. To develop more gene-linked markers in the proximal side of *Pm61*, the 5.3 Mb sequences (713,523,186–718,866,838) of the reference genome corresponding to the *Pm61* flanking markers *Xicsn2* and *Xicsn4* were used as templates to design SSR primer pairs. The 1.5 Mb (713,528,439–715,057,737) genomic sequences extended from marker *Xicsn2* toward *Pm61* was used to design 347 SSR primer pairs (Figure 5C, Supplementary Table S4). The 2.5 Mb (718,854,012–716,351,892) sequences extended from marker *Xicsn4* to *Pm61* were used to design 617 SSR primer pairs (Figure 5C, Supplementary Table S5). Five co-dominant SSR markers, *Xicscx305*, *Xicscx497*, *Xicscx543*, *Xicscx741*, and *Xicscx834* and a dominant SSR marker *Xicscx848*, were polymorphic between the two parents and two DNA bulks, which indicates their possible linkage to *Pm61*.

### 2.7. Construction of the Genetic Linkage Map for Pm61

**Figure 6:**
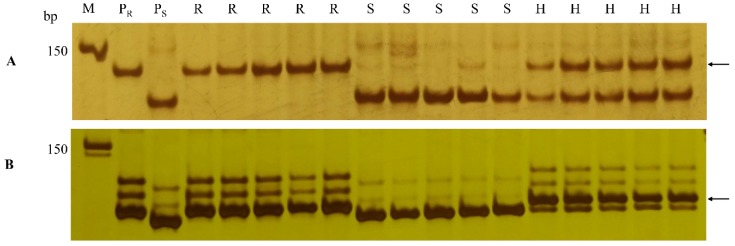
The banding patterns of *Pm61*-linked simple sequence repeat (SSR) markers *Xicscx497* (**A**) and *Xicsx538* (**B**) in the parents and the selected F_2:3_ lines of the Xuxusanyuehuang × Zhongzuo 9504 cross. Lane M, 50 bp DNA ladder; P_R_, Xuxusanyuehuang; P_S_, Zhongzuo 9504; R, homozygously resistant F_2:3_ lines; S, homozygously susceptible F_2:3_ lines; and H, heterozygous F_2:3_ lines. Arrows indicate the polymorphic bands specific for *Pm61*.

The polymorphic markers developed, including two SNP markers (*Xicsn2* and *Xicsn4*), two KASP markers (*Xicsk8* and *Xicsk13*), and six SSR markers (*Xicscx305*, *Xicscx497*, *Xicscx543*, *Xicscx741*, *Xicscx834*, and *Xicscx848*), together with seven polymorphic *Pm61*-linked markers developed in the previous study [35], were used to construct the genetic linkage map after genotyping the F_2:3_ mapping population of XXSYH × Zhongzuo 9504. In this linkage map, *Pm61* was placed in a 0.71 cM genetic interval that corresponded to 0.61 Mb genomic interval (718,257,529–718,866,730) of the genomic region in the reference genome sequence of Chinese Spring. The SSR markers *Xicscx543* and *Xicscx497* were located in the same locus at the proximal side of *Pm61* with genetic distance of 0.47 cM. The SNP marker *Xicsn4* was located in the distal side of *Pm61* with genetic distance of 0.24 cM. The KASP markers *Xicsk8* and *Xicsk13* (Figure 4A,B) and SSR markers *Xicscx497* and *Xicsx538* (Figure 6A,B) produced clear banding patterns and were able to differentiate individuals of the mapping population with distinct phenotypes.

### 2.8. Physical Locations of Pm61, QPm.tut-4A and MlIW30 on Chromosome Arm 4AL

**Figure 7:**
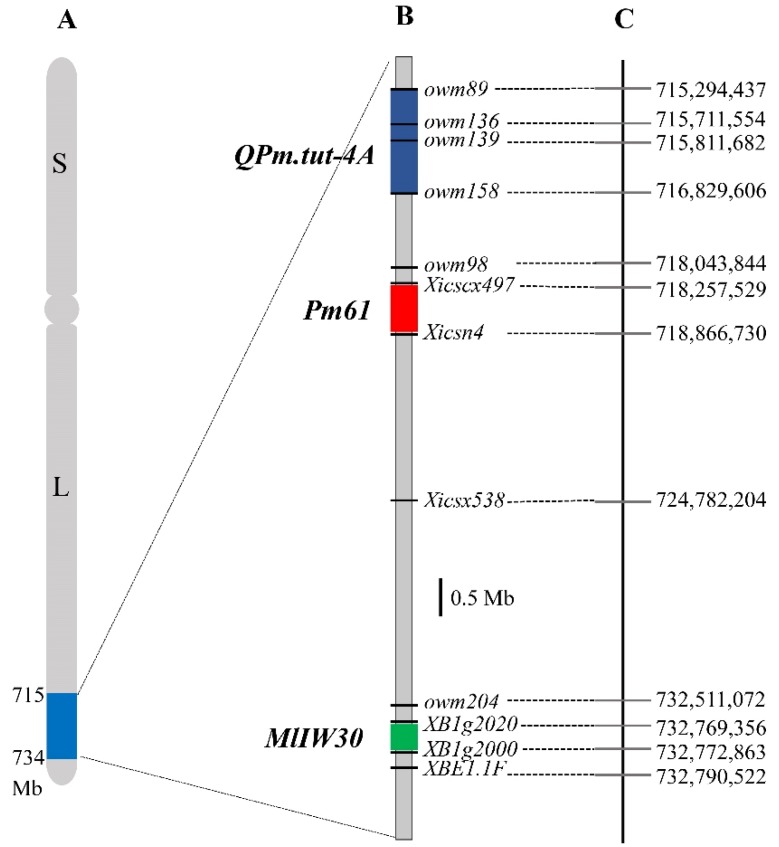
A sketch physical map of chromosome 4A (**A**), comparative analysis of the three *Pm* genes or QTL on chromosome arm 4AL (**B**), and corresponding physical locations in chromosome 4AL in the Chinese Spring reference genome (**C**).

In addition to *Pm61*, a dominant gene *MlIW30* from wild emmer wheat [36] and a QTL *QPm.tut-4A* from *T*. *militinae* Zhuk. et Migusch. [37] were mapped on chromosome arm 4AL. Two *MlIW30*-linked markers, *XB1g2020.2* and *XB1g2070.1*, were linked to *Pm61* using the mapping population of XXSYH × Mingxian 169 [35]. However, only *XB1g2070.1* was linked to *Pm61* with genetic distance of 1.43 cM in the mapping population of XXSYH × Zhongzuo 9504. Polymorphism of 73 markers linked to *QPm.tut*-*4A* [37] were examined against the parents and the contrasting DNA bulks of the XXSYH × Zhongzuo 9504 cross, resulting in 5 polymorphic markers *owm73*, *owm98*, *owm104*, *owm158*, and *owm204* that were linked to *Pm61*. The closest *QPm.tut*-*4A*–linked marker *owm158* to *Pm61* was 0.95 cM from *Pm61*. Based on the physical position on the Chinese Spring reference genome sequence, *Pm61* was located in 0.61 Mb physical interval (718,257,529–718,866,730) between *MlIW30* (21 kb, 732,769,506–732,790,522) and *QPm.tut*-*4A* (1.54 Mb, 715,294,437–716,829,606 bp) (Figure 7).

## 3. Discussion

Using the strategy of BSR-Seq analysis that was performed on the mapping population of XXSYH × Zhongzuo 9504, two *Pm61*-linked SNP markers (*Xicsn2* and *Xicsn4*) and two KASP markers (*Xicsk8* and *Xicsk13*) were developed. Based on the genomic sequences of the Chinese Spring reference genome that flanked the *Pm61* locus, six SSR markers were linked to the target gene. Using these molecular markers, together with previously developed gene-linked markers [35,37], a new saturated genetic linkage map was established, which placed *Pm61* in a 0.71 cM genetic interval corresponding to a 0.61 Mb physical interval (718,257,529–718,866,730) on the terminal region of chromosome 4AL. The closest flanking markers of *Pm61* were *Xicscx497/Xicscx543/owm73/owm98/owm104* and *Xicsn4* with genetic distances of 0.47 and 0.24 cM, respectively. *Pm61* can be detected with the KASP markers *Xicsk8* and *Xicsk13* and SSR markers *Xicscx497* and *Xicsx538*. Compared to the previous study [35], the newly developed genetic linkage map was saturated with more molecular markers especially in the proximal side of *Pm61*. The physical location of *Pm61* in the Chinese Spring reference genome between the two genetic linkage maps with different mapping populations is comparable.

Previously identified *Pm61*-linked markers, *Xicsx79* and *Xicsx436* [35], the only two markers in the proximal side of the target gene, were not polymorphic between the parents XXSYH and Zhongzuo 9504. Therefore, BSR-seq analysis was performed to find more polymorphic markers that flanked *Pm61.* Two SNP markers *Xicsn2* and *Xicsn4* were located on the opposite sides of *Pm61*. The fragments of genomic sequences from the Chinese Spring reference genome corresponding to the two SNP markers were used to develop SSR markers, which produced 6 markers that were linked to *Pm61*. After genetic linkage analysis, all these markers were located in the proximal side of *Pm61*. The newly developed *Pm61*-linked markers are being used to genotype large scale of F_2_ and F_2:3_ population that was derived from XXSYH × Zhongzuo 9504 cross. The saturation of the genetic linkage map for *Pm61* facilitates its fine mapping and ultimately map-based cloning.

The race-specific *Pm* genes inherit either in a dominant mode or in a recessive mode. Many Chinese landraces were identified to possess recessive *Pm* genes, for example, several genes in the *Pm5* locus [4,5,6,8,9,10,11,12], *Pm47* [17], and *PmX* [18]. *Pm61* in XXSYH is also a recessive gene [35]. A *Pm* gene with such a recessive mode of inheritance needs additional generation to allow the expression of resistant phenotype when the target gene is homozygous.

The establishment of the MAS technique for *Pm61* will facilitate its application in developing disease resistant wheat cultivars. The KASP markers *Xicsk8* and *Xicsk13* are able to identify *Pm61* in a high throughput scale. KASP assay is also known as tolerance to DNA quality, low cost and high specificity [38]. However, KASP assay may produce a certain proportion of calling errors (0.7~1.6%) [38] and (8.8% ± 5.5%) missing data [39]. The SSR markers *Xicscx497* and *Xicsx538* are useful to detect *Pm61* using a standard DNA amplification method. Many breeders are able to use PCR-based molecular markers in their routine breeding practices, since such type of molecular markers is cost-effective. The combination KASP assay and PCR analysis can provide a more accurate identification of the target gene during the process of MAS.

Three *Pm* genes or QTL, i.e., *MlIW30*, *Pm61*, and *QPm.tut-4A*, have been detected on a 17.50 Mb genomic region on the terminal part of chromosome arm 4AL (Figure 7). Based on their positions in the Chinese Spring reference genome, *MlIW30* was located in a 21 kb (732,769,506–732,790,522) genomic interval [36]. *QPm.tut-4A* was identified in a 1.54 Mb (715,294,437–716,829,606) genomic interval. *MlIW30* and *QPm.tut-4A* were transferred into common wheat from the wild emmer (*T. turgidum* ssp. *dicoccoides*) and *T. militinae*, respectively [36,37]. *Pm61* is derived from a Chinese landrace and is located in a 0.61 Mb (718,257,529–718,866,730) genomic interval between *MlIW30* and *QPm.uga-4A*. It appears that *Pm61* was located on different genomic intervals as *MlIW30* and *QPm.tut*-*4A*. Isolation and functional analysis of these *Pm* genes will ultimately understand their relationship.

In summary, BSR-seq analysis, in combination with the Chinese Spring reference genome sequence, identified 10 SNP, KASP, and SSR markers, which saturated the genetic linkage map of *Pm61*, especially in the proximal side of the target gene. The development of KASP markers, *Xicsk8* and *Xicsk13*, and SSR markers, *Xicscx497* and *Xicsx538*, allows the detection of *Pm61* in different scales and platforms. Results from this study will facilitate the fine mapping and ultimate map-based cloning, as well as application in breeding and agriculture, of *Pm61* gene.

## 4. Materials and Methods

### 4.1. Plant Materials

Xuxusanyuehuang as the maternal parent was crossed to powdery mildew susceptible winter wheat Zhongzuo 9504 to generate F_1_, F_2_ and F_2:3_ populations for analyzing the inheritance mode of the resistance gene, determining polymorphism of molecular markers, and establishing linkage relationships between polymorphic markers and *Pm61*. Zhongzuo 9504 also served as the susceptible control in the powdery mildew tests and provided the host plants to maintain and increase *Bgt* isolates.

### 4.2. Assessments of Resistance to Powdery Mildew

Thirty *Bgt* isolates collected from Shandong, Shanxi, Beijing, Hebei, and Sichuan wheat producing provinces were used to test responses of XXSYH to powdery mildew. These isolates were subjected to three rounds of single-pustule culture on Zhongzuo 9504 plants prior to inoculation. *Bgt1* from Shandong province was used in phenotyping the F_2:3_ mapping population. At least 15 seeds of each F_2:3_ line and the parents were planted in 8-cm-diameter plastic pots. Inoculation of *Bgt* isolates was conducted when wheat seedlings were at 2-leaf stage. Seedlings were dusted with freshly increased conidiophores, incubated in a dew chamber with 90% relative humidity, and grown in a greenhouse to allow development of powdery mildew symptoms. The conditions of plant growth were set at 20–22 °C/14 °C (day/night) with 16 h light/8 h dark photoperiod. When the disease symptoms were fully developed on the susceptible control plants 15 day after inoculation, symptom scoring was conducted by determining the infection type (IT) of each plant on a 0–4 scale as described previously [40]. Based on the scores of ITs, plants with ITs 0–2 were categorized into the resistant group, and those with ITs 3–4 into the susceptible group.

### 4.3. BSR-Seq Analysis

Based on the phenotypic evaluations, 30 homozygous resistant (IT 1) and 30 homozygous susceptible (IT 4) lines in the F_2:3_ mapping populations of XXSYH × Zhongzuo 9504 were selected to construct the phenotypically contrasting bulks. Each line was represented by a single plant that was free of *Bgt* inoculation. The leaf segments approximately 3 cm long from each plant two-week old were sampled and pooled as the resistant and susceptible bulks for isolating total RNA following the TRIzol protocol (Invitrogen, Carlsbad, CA, USA). RNA-seq analysis was performed in the platform of Illumina HiSeq 2500 in Beijing Novogene Bioinformatics Technology Co. Ltd. (Beijing, China). To remove the adapter sequences and low-quality sequences, raw reeds of generated were subjected to quality control using the software Trimmomatic v0.36 (available online: http://www.sadellab.org/cms/index.php?page=trimmomatic) [41]. The high-quality reads were aligned to the Chinese Spring reference genome sequence v1.0 and its annotation files [25], which was carried using the software STAR v2.5.1b (available online: http://code.google.com/p/rna-star/.) [42], with the mismatch rate of less than 5%. After removing PCR optical duplicates and spliting the mapped reads spanning introns, the unique and confident alignments were used to call SNP variants using “HaplotypeCaller” module in the software GATK v3.6 (available online: http://www. broadinstitute.org/gsa/wiki/index.php/The_Genome_Analysis_Toolkit) [43]. A Fish Exact Test (FET) and allele frequency for each variant and allele frequency difference (AFD) between the resistant and susceptible bulks were used to identify SNP variants. The SNP variants with *P* < 1 × 10^−10^ and AFD > 0.6 were regarded as candidate SNP linked to the target gene and used as templates for developing SNP markers.

### 4.4. DNA Isolation, Amplification and Electrophoresis

The leaf tissues of each F_2:3_ line were used for DNA extraction after disease resistance test following the CTAB protocol [44]. DNA concentration was determined using the Napdrop One (Thermo Fisher Scientific Inc, Madison, WI, USA) and was adjusted to 50 ng·mL^−1^. DNA bulks with resistant or susceptible phenotype were constructed by pooling equal amounts of DNA from 10 resistant and susceptible F_2:3_ lines each. DNA was amplified in a Biometra T3000 Thermocycler (ABI, New York, NY, USA). Each reaction mixture (10 μL) was composed of 5 μL mixture (including *Taq* polymerase, dNTPs, and 10× PCR buffer and Mg^2+^), 2 μL ddH_2_O, 1 μL DNA, and 1 μL 10 μM each of the forward and reverse primers. The profile of DNA amplification was set at 98 °C for 3 min; 35 cycles of 98 °C for 10 s, 55 °C–60 °C (depending on the specific primers) for 10 s and 72 °C for 25 s; 72 °C for 10 min. Products amplified were separated by 2% agarose gel or 8% non-denaturing polyacrylamide gel (Acr:Bis = 29:1).

### 4.5. Development and Validation of SNP Markers

Approximately 3 kb sequences extracted from the RefSeqv1.0 Chinese Spring genome sequence [25] containing the candidate SNP linked to the target gene were used as queries in searching the Chinese Spring whole genome assembly (available online: https://urgi.versailles.inra.fr/) to acquire the homologous scaffolds. The sequences containing candidate SNP that corresponded to the above homologous scaffolds were used as templates to design SNP primers on the GSP website (available online: https://probes.pw.usda.gov/GSP/) [45]. The primers designed contained at least one variant site at the 3′ ends and were anticipated to amplify products in a range of 300~1000 bp in length. The polymorphism of SNP primers was validated in the parents and the two DNA bulks by analyzing the sequences of the amplicons (Invitrogen Trading Co., Ltd., Shanghai, China), and the polymorphic SNP primers were used to genotype the F_2:3_ mapping population.

### 4.6. Conversion of SNP Markers to KASP Markers

The polymorphic SNP primers were converted to KASP markers using the PolyMarker software (available online: http://polymarker.tgac.ac.uk) [46]. Each KASP reaction was carried out using a 5 μL reaction mixture consisting of 2.2 μL DNA, 2.5 μL 2× KASP master mix, 0.056 μL primer mix (12 mM of each allele-specific primer and 30 mM of the common primer), 0.039 μL Mg^2+^, and 0.205 μL ddH_2_O. Amplification was performed in the ABI 7500 device (Applied Biosystems, Foster City, CA, USA) with the program of 94 °C for 15 min; 35 cycles of 94 °C for 20 s and 60 °C for 1 min. The FLUOstar Omega microplate reader (BMG Labtech, Durham, NC, USA) was used to read the green (521 nm) and pink (556 nm) fluorescence signals at 25 °C for 2 min after the reactions were completed. The fluorescence signals were transformed into different genotypes, i.e., FAM homozygotes, HEX homozygotes, and FAM/HEX heterozygotes using the Klustering Caller software (available online: http://www.lgcgroup.com/).

### 4.7. Development of SSR Markers

The sequences of *Pm61*-linked SNP markers designed were used to search for the Chinese Spring reference genome sequence v1.0 [25]. The corresponding genomic sequences were used as templates to design SSR markers with BatchPrimer3 software (available online: https://wheat.pw.usda.gov/demos/batchprimer3). Polymorphism of SSR markers were examined using the parents and the contrasting DNA bulks. DNA amplification and visualization of banding patterns were carried out as described previously [35]. The polymorphic markers were used to establish a genetic linkage map of *Pm61* with the F_2:3_ mapping population.

### 4.8. Construction of High-Density Genetic Linkage Map

The *Pm61*-linked markers, including previously developed SSR markers and the SNP markers, KASP markers, and SSR markers developed in present studies [35,37], were used to construct the high-density genetic linkage map of *Pm61* using the software Mapdraw v.2.1 (Huazhong Agricultural Sciences, Wuhan, China) [47]. The genetic distance was measured by the Kosambi function. Linkage relationship between markers and *Pm61* was established with the software Mapmaker/Exp Version 3.0b and a logarithm of the odd ratio (LOD) score threshold of 3.0 [48].

## Figures and Tables

**Table 1:** Infection types of Xuxusanyuehuang (XXSYH) and Zhongzuo 9504 to 30 *Blumeria graminis* f. sp. *tritici* (*Bgt*) isolates from different provinces of China.

*Bgt* Isolate	XXSYH	Zhongzuo 9504	Province
1	1	4	Shandong
2	1	3	Shandong
3	1	3	Shandong
4	2	3	Shandong
5	1	3	Shandong
6	2	3	Shandong
7	3	3	Shandong
8	3	4	Shanxi
9	2	3	Shanxi
10	0;	4	Shanxi
11	3	3	Shanxi
12	2	3	Shanxi
13	1	3	Beijing
14	1	3	Beijing
15	1	3	Beijing
16	3	4	Beijing
17	1	3	Hebei
18	3	3	Hebei
19	0;	3	Hebei
20	3	4	Hebei
21	3	3	Hebei
22	1	3	Hebei
23	1	3	Hebei
24	3	3	Hebei
25	1	3	Hebei
26	2	4	Hebei
27	3	3	Hebei
28	2	4	Hebei
29	3	3	Sichuan
30	1	4	Sichuan
31	2	3	Sichuan

Note: the infection type on leaves was rated on a 0–4 scale for determine the response of wheat genotypes to powdery mildew, where 0 = immune, no symptom, 0; = hypersensitive necrotic flecks, 1 = highly resistant, necrosis with low sporulation, 2 = moderately resistant, necrosis with moderate sporulation, 3 = moderately susceptible, moderate to high sporulation, and 4 = highly susceptible, no necrosis with full sporulation.

## Supplementary Materials

Supplementary materials can be found at http://www.mdpi.com/1422-0067/20/3/750/s1. Table S1 Sequencing quality assessment details; Table S2 A list of SNP marker primers used in this study; Table S3 A list of KASP marker primers used in this study; Table S4 A list of 347 SSR primer pairs based on the 1.5 Mb sequences extended from marker *Xicsn2* toward *Pm61*; Table S5 A list of 617 SSR primer pairs based on the 2.5 Mb sequences extended from marker *Xicsn4* toward *Pm61*.
